# Supplementary material for: Prevalence of Campylobacter and non-typhoidal Salmonella along broiler chicken production and distribution networks, Northern Vietnam
Source: PLoS Negl Trop Dis. 2025 Nov 5;19(11):e0013615. doi: 10.1371/journal.pntd.0013615 (PMC12637993; doi:10.1371/journal.pntd.0013615)
Supplement: S1 Table — (DOCX) [file pntd.0013615.s001.docx]

**S1 Table. Number of selected sites and the number of chickens sampled by different site types.**

| Type | Farm | Retail | Wholesale | Slaughterhouse | Slaughter point | Total |
| --- | --- | --- | --- | --- | --- | --- |
| Number of selected sites by province* | | | | | | |
| Bac Giang | 17 | 4 | 3 | 3 | 5 | 32 |
| Ha Noi | 20 | 4 | 7 | 3 | 5 | 39 |
| Hai Duong | 9 | 4 | 1 | 0 | 5 | 19 |
| Quang Ninh | 4 | 4 | 0 | 0 | 4 | 12 |
| *Total* | *50* | *16* | *11* | *6* | *19* | *102* |
| Number of chickens sampled by breed* | | | | | | |
| Slow-growing broilers | 250 | 80 | 55 | 25 | 90 | 500 |
| Fast-growing broilers | 0 | 0 | 15 | 15 | 35 | 65 |
| *Subtotal* | *250* | *80* | *70* | *40* | *125* | *565* |

*The table presents two different metrics: the upper section shows the number of selected sites across various provinces, while the lower section details the number of chickens sampled across different breeds.
