# Supplementary figures and images for: Prevalence of Campylobacter and non-typhoidal Salmonella along broiler chicken production and distribution networks, Northern Vietnam
Source: PLoS Negl Trop Dis. 2025 Nov 5;19(11):e0013615. doi: 10.1371/journal.pntd.0013615 (PMC12637993; doi:10.1371/journal.pntd.0013615)

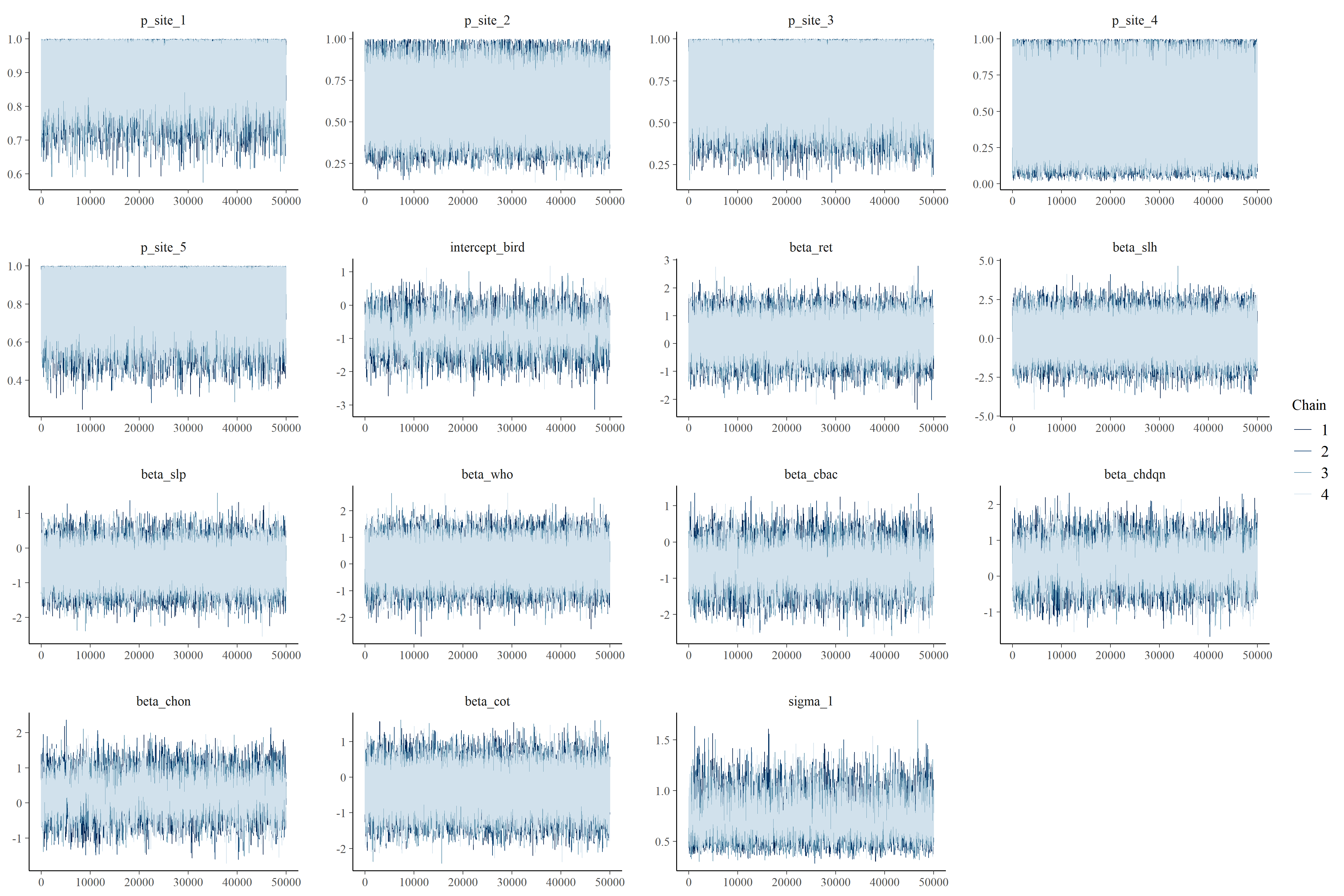

Supplement: S1 Fig — This figure shows the trace plots (n chains = 4, n iteration = 50000) of the selected C. coli model, which displayed tight, consistent horizontal bands, indicating no visual signs of non-convergence within the chains. The parameters p_site_1, p_site_2, p_site_3, p_site_4, and p_site_5 represent the C. coli contamination probabilities for farms, retail markets, wholesale markets, slaughterhouses, and slaughter points, respectively. The parameter intercept_bird represents the baseline probability of a broiler testing positive for C. coli at a contaminated site, assuming no effect from site type and province of supply areas. The fixed-effect coefficients beta_ret, beta_slh, beta_slp, and beta_who quantify the effects of retail markets, slaughterhouses, slaughter points, and wholesale markets on the probability of a broiler testing positive for C. coli at a contaminated site, respectively. The fixed-effect coefficients beta_cbac, beta_chdqn, beta_chon, and beta_cot quantify the effects of supply areas—Bac Giang, Hai Duong/Quang Ninh, Ha Noi, and other provinces—on the probability of a broiler testing positive for C. coli at a contaminated site, respectively. The parameter sigma_1 means the variance of the random intercept. (TIFF) [file pntd.0013615.s002.tiff]

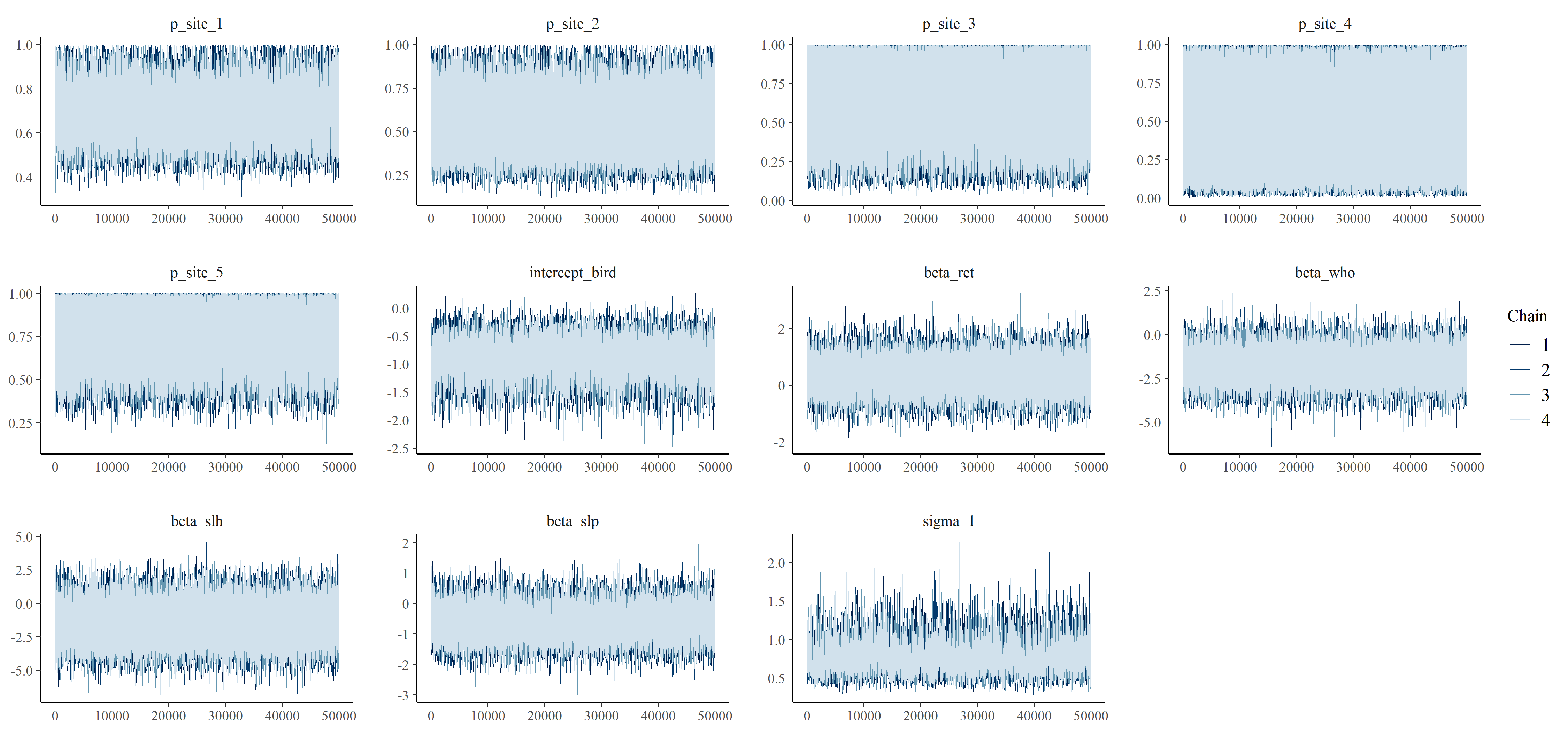

Supplement: S2 Fig — This figure shows the trace plots (n chains = 4, n iteration = 50000) of the selected C. jejuni model, which displayed tight, consistent horizontal bands, indicating no visual signs of non-convergence within the chains. The parameters p_site_1, p_site_2, p_site_3, p_site_4, and p_site_5 represent the C. jejuni contamination probabilities for farms, retail markets, wholesale markets, slaughterhouses, and slaughter points, respectively. The parameter intercept_bird represents the baseline probability of a broiler testing positive for C. jejuni at a contaminated site, assuming no effect from site type. The fixed-effect coefficients beta_ret, beta_who, beta_slh, and beta_slp quantify the effects of retail markets, wholesale markets, slaughterhouses, and slaughter points on the probability of a broiler testing positive for C. jejuni at a contaminated site, respectively. The parameter sigma_1 means the variance of the random intercept. (TIFF) [file pntd.0013615.s003.tiff]

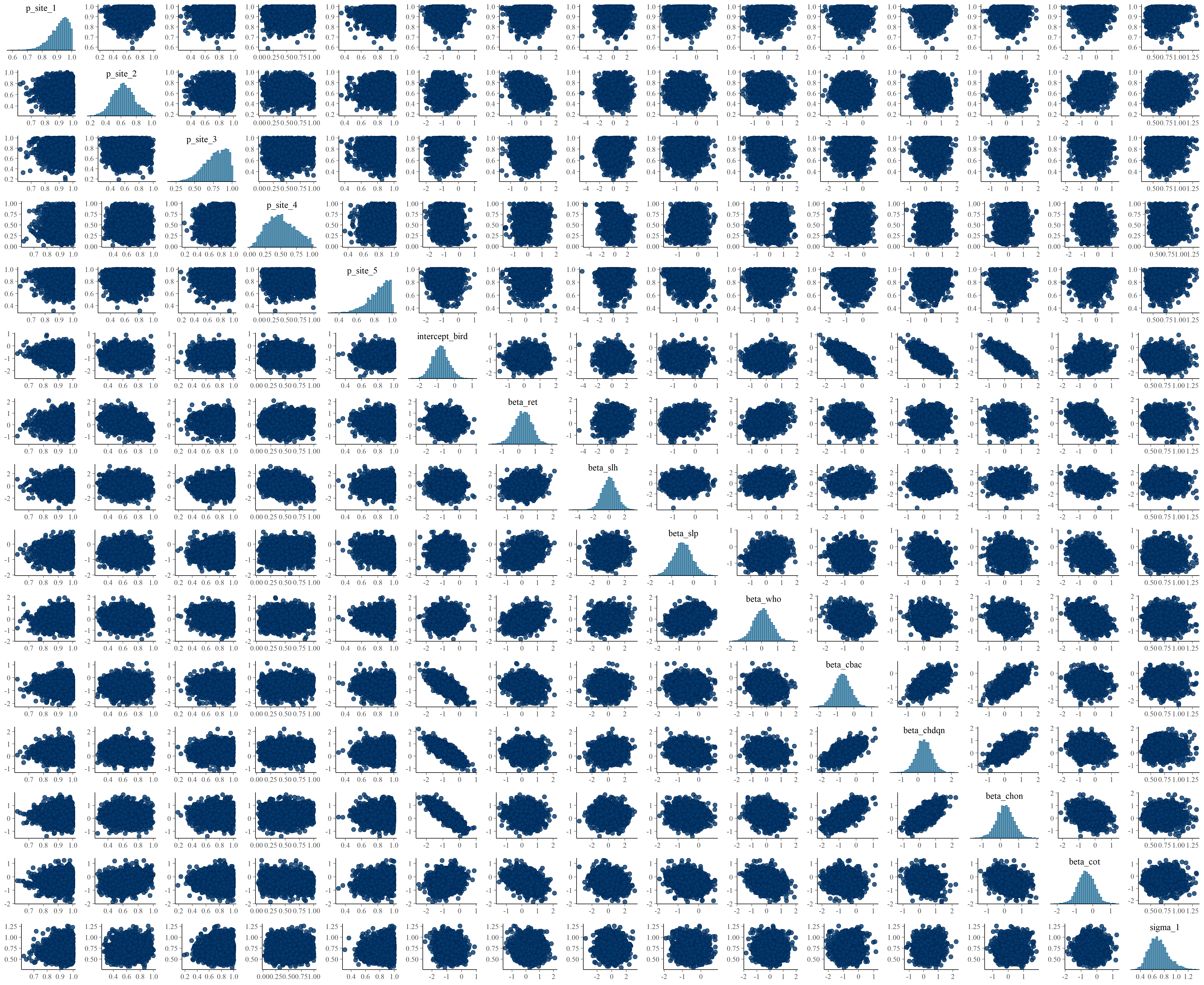

Supplement: S3 Fig — This figure shows the pairs plot of the selected C. coli model, which showed the distribution of each parameter’s posterior values and its interactions with other parameters. The parameters p_site_1, p_site_2, p_site_3, p_site_4, and p_site_5 represent the C. coli contamination probabilities for farms, retail markets, wholesale markets, slaughterhouses, and slaughter points, respectively. The parameter intercept_bird represents the baseline probability of a broiler testing positive for C. coli at a contaminated site, assuming no effect from site type and province of supply areas. The fixed-effect coefficients beta_ret, beta_slh, beta_slp, and beta_who quantify the effects of retail markets, slaughterhouses, slaughter points, and wholesale markets on the probability of a broiler testing positive for C. coli at a contaminated site, respectively. The fixed-effect coefficients beta_cbac, beta_chdqn, beta_chon, and beta_cot quantify the effects of supply areas—Bac Giang, Hai Duong/Quang Ninh, Ha Noi, and other provinces—on the probability of a broiler testing positive for C. coli at a contaminated site, respectively. The parameter sigma_1 means the variance of the random intercept. (TIFF) [file pntd.0013615.s004.tiff]

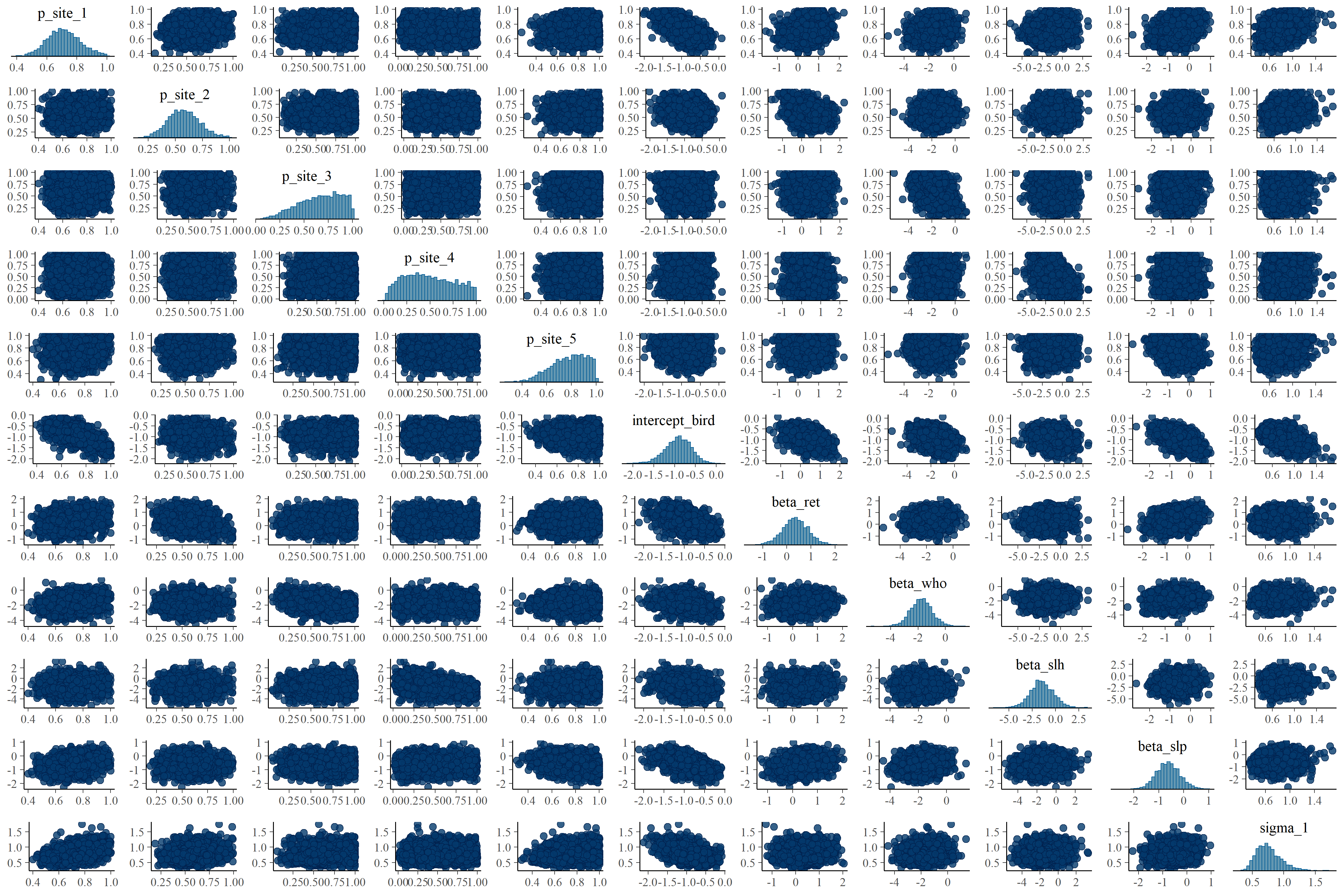

Supplement: S4 Fig — This figure shows the pairs plot of the selected C. jejuni model, which showed the distribution of each parameter’s posterior values and its interactions with other parameters. The parameters p_site_1, p_site_2, p_site_3, p_site_4, and p_site_5 represent the C. jejuni contamination probabilities for farms, retail markets, wholesale markets, slaughterhouses, and slaughter points, respectively. The parameter intercept_bird represents the baseline probability of a broiler testing positive for C. jejuni at a contaminated site, assuming no effect from site type. The fixed-effect coefficients beta_ret, beta_who, beta_slh, and beta_slp quantify the effects of retail markets, wholesale markets, slaughterhouses, and slaughter points on the probability of a broiler testing positive for C. jejuni at a contaminated site, respectively. The parameter sigma_1 means the variance of the random intercept. (TIFF) [file pntd.0013615.s005.tiff]

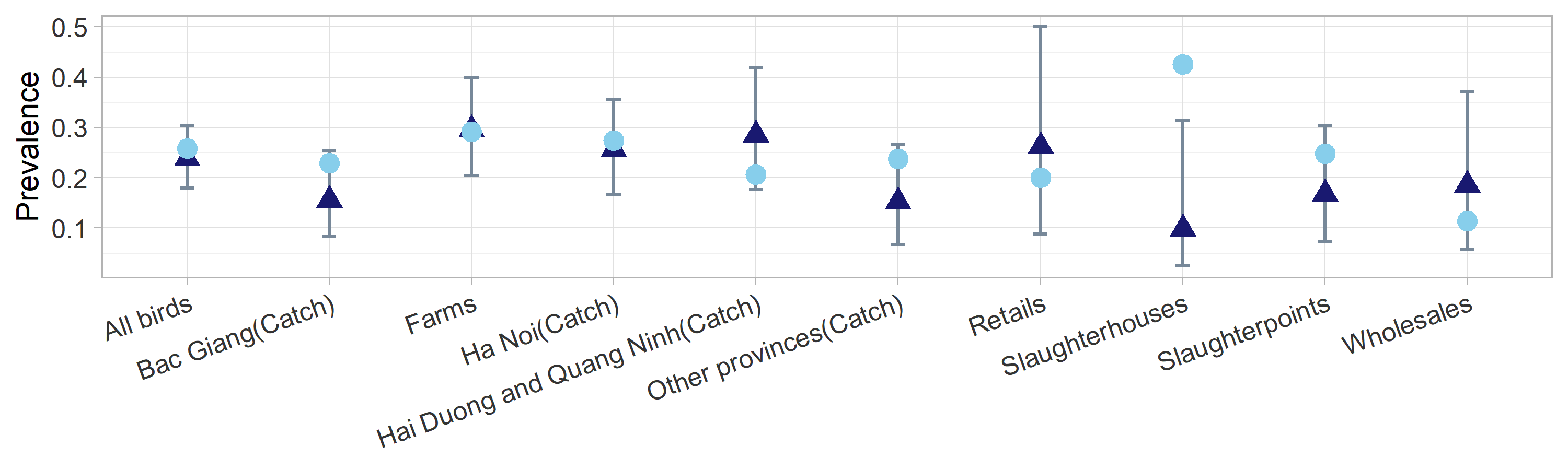

Supplement: S5 Fig — (TIFF) [file pntd.0013615.s006.tiff]

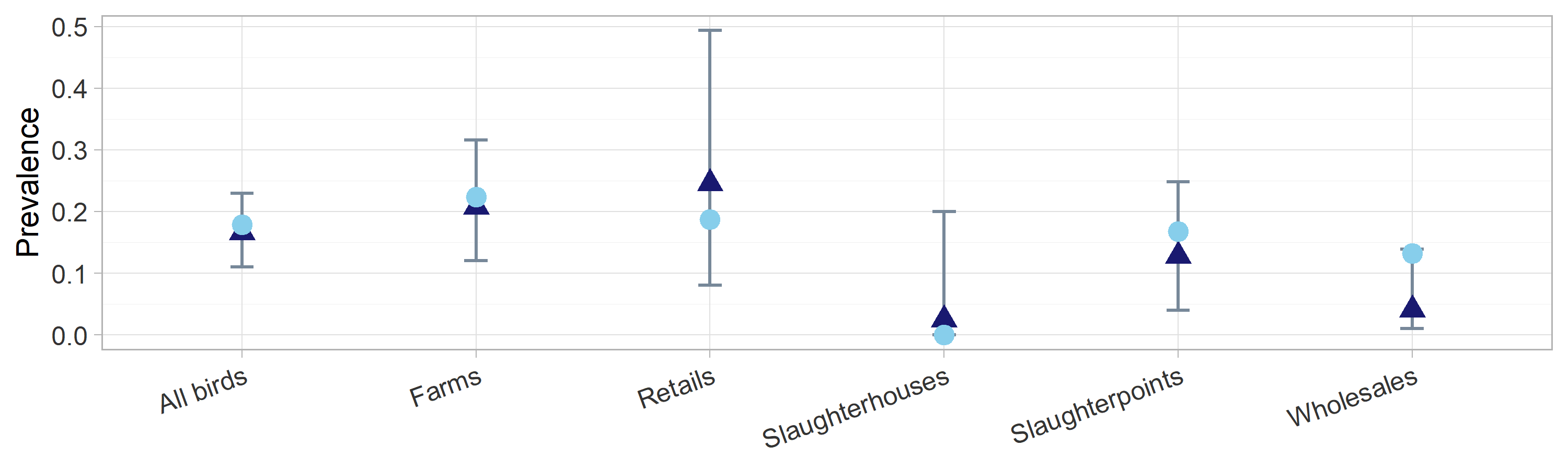

Supplement: S6 Fig — (TIFF) [file pntd.0013615.s007.tiff]

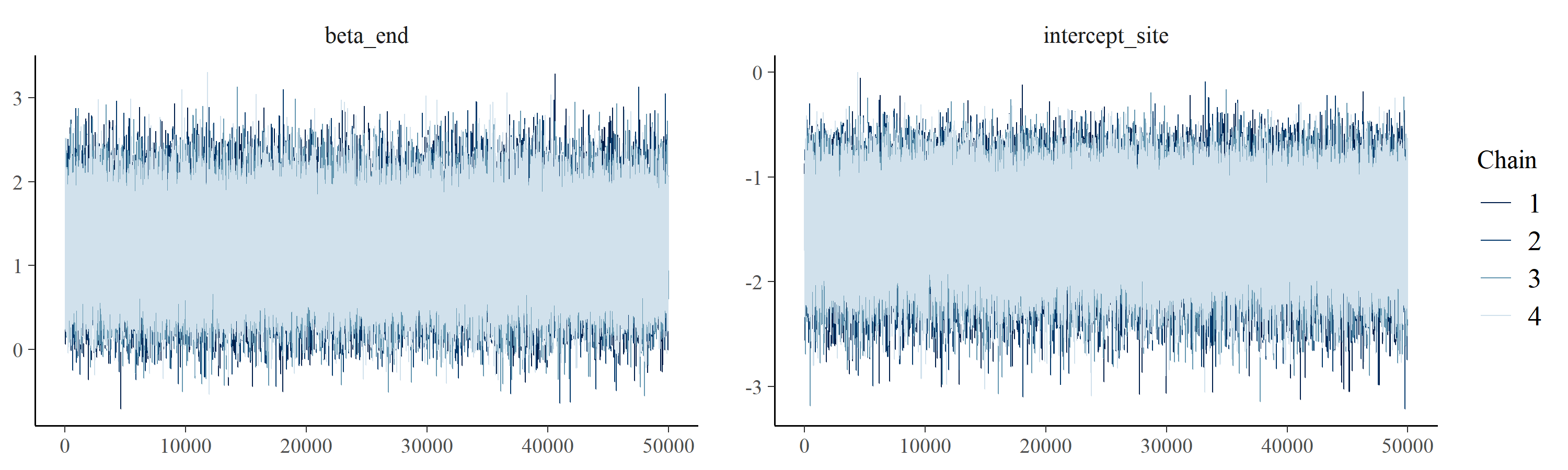

Supplement: S7 Fig — This figure shows the trace plots (n chains = 4, n iteration = 50000) of the selected NTS model, which displayed tight, consistent horizontal bands, indicating no visual signs of non-convergence within the chains. The fixed-effect coefficient beta_end quantifies the effect of endpoints on the probability of a site being contaminated with NTS. The parameter intercept_site represents the baseline NTS contamination probability of a site when site type has no effect. (TIFF) [file pntd.0013615.s008.tiff]

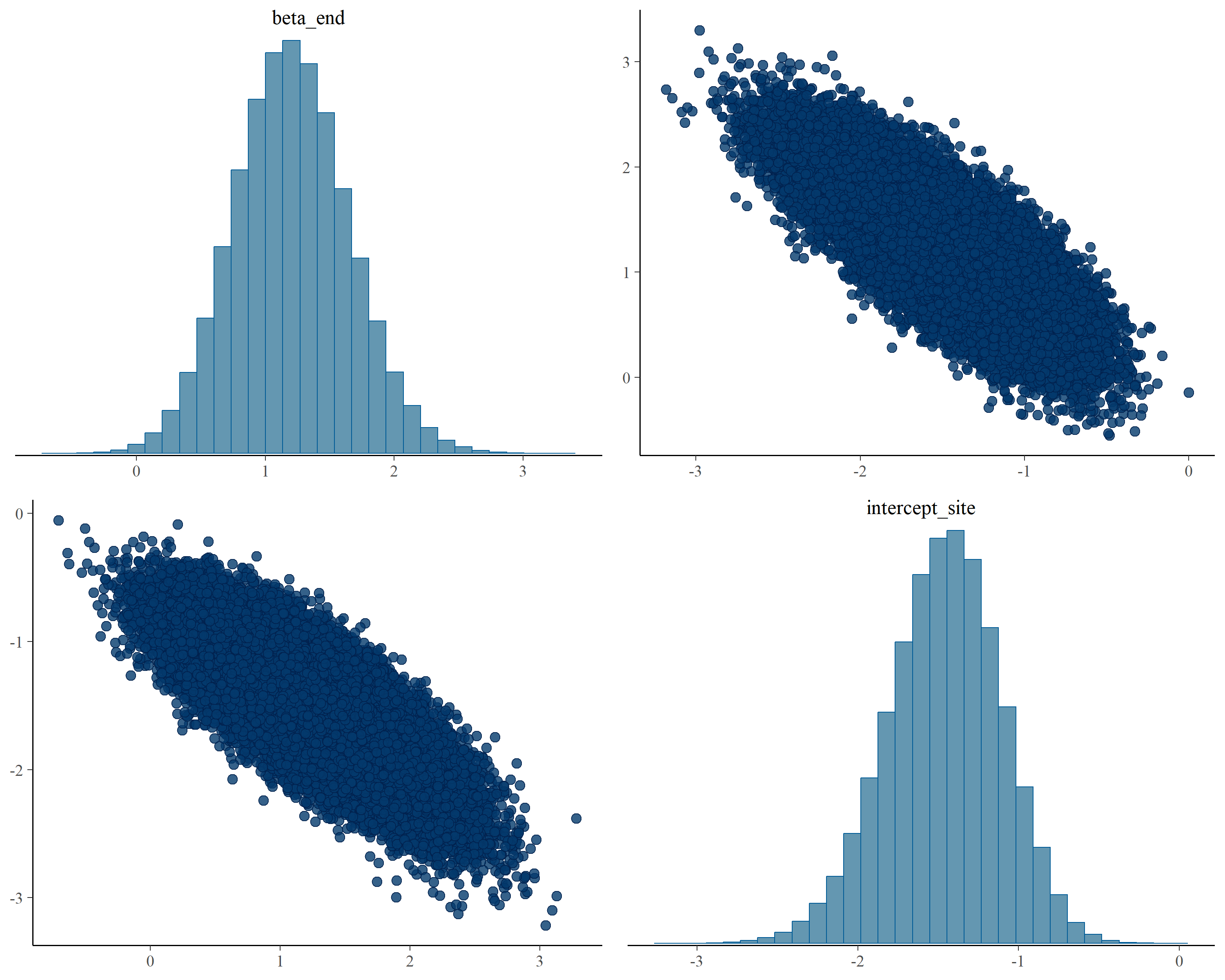

Supplement: S8 Fig — This figure shows the pairs plot of the selected NTS model, which showed the distribution of each parameter’s posterior values and its interactions with other parameters. The fixed-effect coefficient beta_end quantifies the effect of endpoints on the probability of a site being contaminated with NTS. The parameter intercept_site represents the baseline NTS contamination probability of a site when site type has no effect. (TIFF) [file pntd.0013615.s009.tiff]

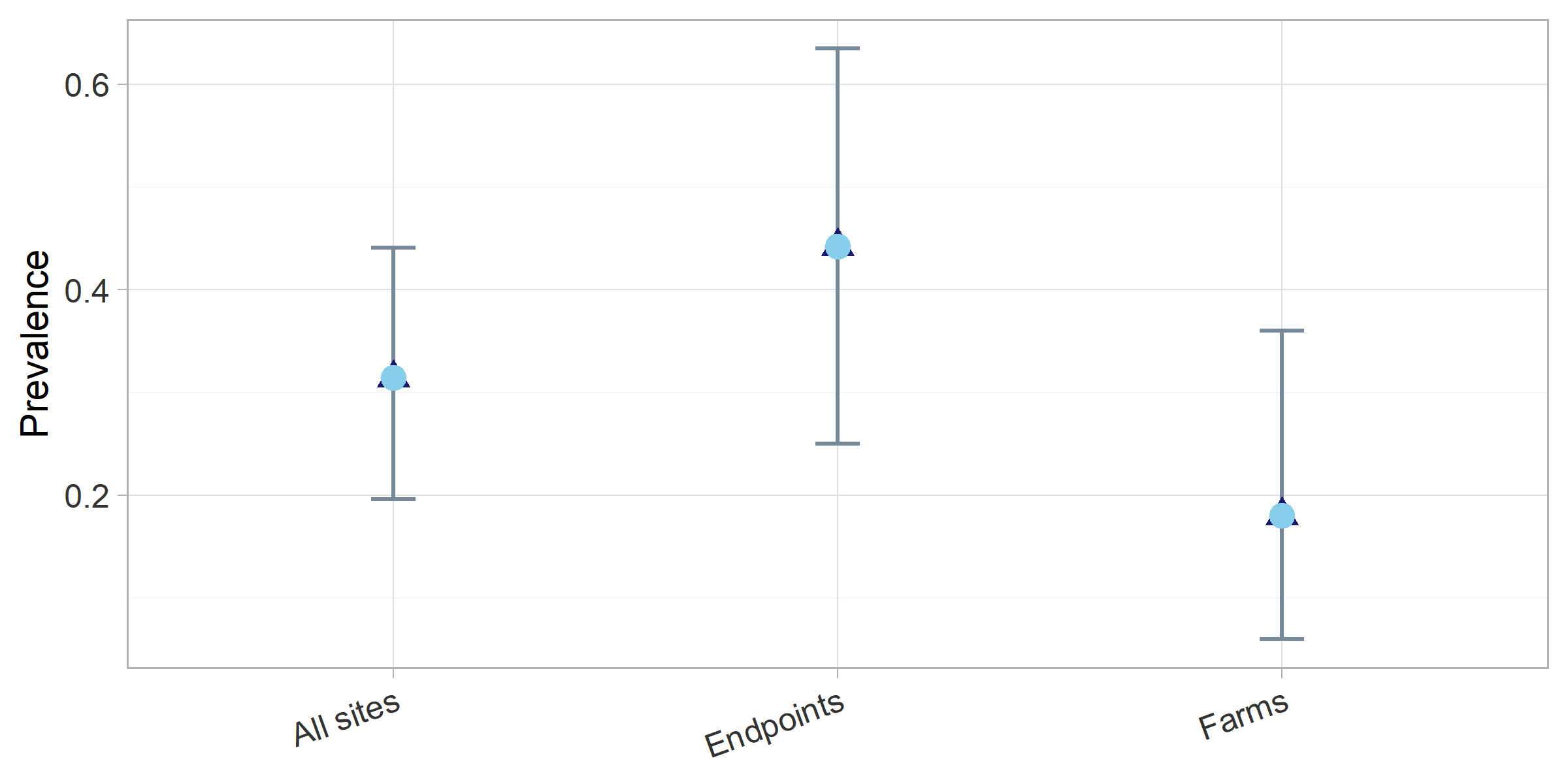

Supplement: S9 Fig — (TIFF) [file pntd.0013615.s010.tiff]
