# Supplementary material for: Prevalence of Campylobacter and non-typhoidal Salmonella along broiler chicken production and distribution networks, Northern Vietnam
Source: PLoS Negl Trop Dis. 2025 Nov 5;19(11):e0013615. doi: 10.1371/journal.pntd.0013615 (PMC12637993; doi:10.1371/journal.pntd.0013615)
Supplement: Attachment — Submitted filename: Review 1_response letter.docx [file pntd.0013615.s013.docx]

July 29, 2025

PLoS Neglected Tropical Diseases

Re: Decision on Manuscript PNTD-D-25-00373R1

Dear editor and reviewers:

We would like to submit a revised version that takes into account editor’s and reviewers’ comments. Here is a detailed list of our responses in blue to the comments and a description of the changes we made in the manuscript. The line numbers refer to the track change version. Revised versions of the manuscript with and without track changes are attached. All co-authors agree with the edits.

We hope that the revised manuscript will be suitable for publication in PLoS Neglected Tropical Diseases.

Yours sincerely,

Dr. Anne Conan

# Comments from Editors:

- **Organization of the methods section of the manuscript. Please make sure that the selection of the province wards, sampling points and poultry samples are explained and justified. Currently, its overall cumbersome to understand the selection and inclusion process.**

**Answer:** The authors value the editor's feedback on our manuscript. We changed the section order in the Materials and Methods section. Now the order is: 'Study Area and Study Design', 'Site Selection', 'Chicken and Environment Sampling', 'Isolation and Identification of *Campylobacter* spp. and NTS', 'Serotyping of Isolated NTS', 'Bayesian Analysis', 'Ethics Statement'. We also completed the sections, Study Area and Study design and Site Selection to clarify the selection and inclusion process following Reviewer 1 comments.

- **The methods mention the use of an informed consent. Please incorporate an Ethics section in the manuscript.**

**Answer:** We moved our Ethics Statement from the end of the manuscript to the method section. It is now available on lines 297-301:

"*This study was approved by the National Institute of Veterinary Research (Vietnam) (020-433/DD-YTCC) and the Royal Veterinary College (UK) Ethics and Welfare Committee (URN: 2020 1983-3). All participants informedly agreed to participate in the study. Oral consent was obtained from the farmer to sample their chickens*."

- **Please explain how many colonies compatible with Campylobacter and NTS were selected for processing. Please explain how the quality of the NTS genomes was assessed and where are genomes made available to the public.**

**Answer:** Our protocol was to subculture and test five colonies per sample, a balance between sensitivity and cost. We specified on line 207.

*"Five colonies from these plates were screened by Gram-stain and wet mount for typical Campylobacter morphology and motility."*

Moreover, for quality assessment for NTS genome, it is described lines 238-241. We removed the sub section title ('Analysis of WGS data') to avoid mis leading the reader.

Finally, the analysis of the genomes of *Campylobacter* and *Salmonella* will be the focus of two other peer-reviewed publications including cross-country data and will be made available to the public at publication time. We slightly changed the objective description to clarify the aim of the current paper.

"*This study aimed to estimate the prevalence of Campylobacter spp. (C. coli and C. jejuni) and NTS in broiler production and distribution networks (PDNs) using a Bayesian approach. It also investigated the NTS serotypes circulating in these PDN*" (lines 55–56)

"*NTS serotypes circulating in these PDNs were also identified*" (line 132)

- **The methods related to the bioinformatic analysis of NTS is limited. Major editions are required to ascertain the analysis process is reproducible (with reads and genomes made publicly available). For example, what CGE tools were utilized and for what purpose?**

**Answer:** Thank you for your comment. This manuscript primarily focuses on epidemiological investigation rather than detailed genomic characterization. Our bioinformatic analyses description was intentionally limited to essential steps relevant to the secondary objective, i.e. to determine the serotype of NTS isolates.

We recognize that genomic analyses, such as antimicrobial resistance gene profiling, phylogenetic analysis, and detailed genomic characterization, would provide valuable insights. However, such analyses are outside the scope of this epidemiological study. Moreover, these detailed genomic analyses are currently underway as part of a wider cross-country analysis that focuses on genomic surveillance. Detailed methodology, analyses, and discussion will be separately reported in an upcoming manuscript, where the raw sequencing reads and assembled genomes from this study will be deposited in the publicly accessible Pathogen Genomics database, with BioProject accession numbers clearly stated upon publication of that comprehensive genomic manuscript.

- **qPCR methods are missing. Please specify Campylobacter genus primer stated in methods but not visualized in table.**

**Answer:** qPCR was part of the standard quality control for NTS sequencing library preparation. The assay was performed using the KAPA Library Quantification Kit, specifically designed for Illumina sequencing libraries, as recommended by the manufacturer. We clarified:

*" Library concentrations were normalized to 4 nM and validated by qPCR on a QuantStudio-3 real-time PCR system (Applied Biosystems) using the Kapa library quantification kit for Illumina platforms as recommended by the manufacturer (Kapa Biosystems)."* (lines 232-235)

For the PCR of *Campylobacter*, the *C. jejuni* 23S-based primers are used to identify *Campylobacter* species. The other primer sets are the *hipO* gene specific to *C. jejuni* and the *glyA* gene specific to *C. coli*, respectively. We specified in Table 1.

- **Results, Discussion, Conclusion and Table/Figure concerns have already been highlighted by the reviewers.**

**Answer:** Thank you for the highlights. Reviewer comments are addressed below.

# Reviewer comments

## Methods

### Reviewer #1:

- **Are the objectives of the study clearly articulated with a clear testable hypothesis stated? Partially. While the study’s objectives are generally described, they are not fully comprehensive. Specifically, the molecular characterization of isolates via sequencing—an important component of the study—is not included in the stated objectives at the end of the Introduction. The authors should revise the objectives to fully capture the scope of the work and clearly articulate the testable hypothesis.**

**Answer:** Thank you very much for this insightful comment. We have revised our stated objectives both in the abstract and at the end of the Introduction to explicitly include the serotype determination of NTS.

"*This study aimed to estimate the prevalence of Campylobacter spp. (C. coli and C. jejuni) and NTS in broiler production and distribution networks (PDNs) using a Bayesian approach. It also investigated the NTS serotypes circulating in these PDN*" (lines 55–56)

"*NTS serotypes circulating in these PDNs were also identified*" (lines 132)

- **Is the study design appropriate to address the stated objectives?
  Yes. The study employs a cross-sectional design with sampling across multiple stages of the broiler chicken supply chain. This is appropriate for determining prevalence and identifying risk factors along the production and distribution network. However, some aspects of the site selection and stratification require clarification to fully understand the sampling logic and ensure representativeness.**

**Answer:** Thank you for pointing this out. We have moved the study design to the first section of the Materials and Methods 'Study area and study design' (lines 142-152) and described the details of site selection and stratification in the Site Selection section. It is now available on lines 162-176.

*“For endpoints’ selection, a stratified cluster sampling design was adopted, with a stratification by provinces. First inclusion criterion was set as endpoints supplying urban areas. Retail markets and slaughter points fitting this criterion were located in urban areas or around urban centers (Live bird markets are prohibited in Ha Noi’s urban centre), while wholesale markets and slaughterhouses were located in peri-urban or rural areas). Meanwhile, slaughter points needed to process at least 30 chickens a day to ensure access to five chickens to sample. In the four provinces, lists of eligible endpoints (markets and slaughterhouses located in urban and peri-urban areas) were initially compiled through consultation with province and district-level Department of Animal Health (DAH) officers, and stakeholder interviews in visited markets and slaughterhouses. Finally, due to their small numbers, all eligible wholesale markets (N = 11) and slaughterhouses (N = 6) that processed slow-growing broiler chickens in the four study provinces were recruited. Retail markets (N = 16) and slaughter points (N = 19) were selected through stratified cluster sampling: four to five wards were randomly selected from each province. In each ward, one retail and one slaughter point were chosen based on the presence of slow-growing broilers. Fast-growing broilers were sampled when available on the site (S1 Table).”*

- **Is the population clearly described and appropriate for the hypothesis being tested?
  Partially. The population under study—broiler chickens from different supply chain stages in Vietnam—is appropriate. However, details about the number and distribution of sites per type (e.g., slaughterhouses, markets) are not clearly presented in the Methods section and only become evident later in the results and tables. This could lead to confusion about how the sampling population was defined and selected.**

**Answer:** We thank the reviewer for the comment; we now have the number of selected endpoints presented as a table in the S1 Table.

S1 Table. Number of selected sites and the number of chickens sampled by different site types.

| Type | Farm | Retail | Wholesale | Slaughterhouse | Slaughter point | Total |
| --- | --- | --- | --- | --- | --- | --- |
| Number of selected sites by province* | | | | | | |
| Bac Giang | 17 | 4 | 3 | 3 | 5 | 32 |
| Ha Noi | 20 | 4 | 7 | 3 | 5 | 39 |
| Hai Duong | 9 | 4 | 1 | 0 | 5 | 19 |
| Quang Ninh | 4 | 4 | 0 | 0 | 4 | 12 |
| *Total* | *50* | *16* | *11* | *6* | *19* | *102* |
| Number of chickens sampled by breed* | | | | | | |
| Slow-growing broilers | 250 | 80 | 55 | 25 | 90 | 500 |
| Fast-growing broilers | 0 | 0 | 15 | 15 | 35 | 65 |
| *Subtotal* | *250* | *80* | *70* | *40* | *125* | *565* |

*The table presents two different metrics: the upper section shows the number of selected sites across various provinces, while the lower section details the number of chickens sampled across different breeds.

- **Is the sample size sufficient to ensure adequate power to address the hypothesis being tested?
  Unclear. While the study includes a large number of samples and reports meaningful prevalence estimates, no justification for the sample size or power calculation is provided. Including this information would strengthen the methodological rigor and support the validity of the findings.**

**Answer:** Sample size was not calculated prior to the study. We have clarified this point in lines 142-144 ("*The project aimed to assess the transmission of pathogens between farms and PDN endpoints; the sample size needed to be defined based on the expected size of targeted bacterial populations, which were unknown. Number of sites to select was set to sample chickens as widely as possible."*). We chose to use Bayesian inference, which remains valid even with a low sample size. As suggested by the reviewer, we conducted a retrospective power analysis based on the analysis goal to assess a meaningful difference in NTS contamination and *Campylobacter* infection between the different factors. The power analysis is now described in both the Materials and Methods section and the Results. Interpretation of the power was also added in the discussion.

"*Finally, we conducted Bayesian retrospective power analyses for Campylobacter and NTS models to evaluate the models' ability to detect meaningful differences of 10 % in prevalence and contamination probability between the different explanatory variables. The HDI of the estimated power was also computed [37]."* (lines 293-296)

[37] Kruschke JK. Doing bayesian data analysis: A tutorial with R and BUGS. New York, NY, USA: Academic Press; 2010.

"*The ability of the model to detect meaningful differences in Campylobacter prevalence was estimated to have a power of 68% (95% HDI 59-77%) for farm vs endpoints at the site level (contamination), 35% (95% HDI 26-45%) at the bird level, 28% (95% HDI 20-75%) for slow-growing vs. fast-growing broiler and 61% (95% HDI 51-70%) for province*" (lines 387-390)

" *The estimated power of the NTS model to detect meaningful differences of contamination between farms and endpoints was 79% (95% HDI 70-86%)."* (lines 431-432)

*"Given the small sample size, our estimation, based on the Bayesian framework with weakly informative priors, might introduce limited bias in posterior estimates [37] and led to a relatively low power for some variables. The absence of a difference between type of birds may then be due to this low statistical power."* (lines 526-529)

- **Were correct statistical analyses used to support conclusions?
  Yes, with minor concerns. The use of Bayesian models and molecular analyses is appropriate and robust. However, the order of presentation of the methods does not align with the results, which can make interpretation difficult. Reorganizing the methods to match the results flow would improve clarity.**

**Answer:** Thank you for pointing this out. However, for clarity and following a comment further, we decided to reorganize the results to match the method flow: 'Descriptive Results for *C. coli* and *C. jejuni'*, 'Descriptive Results and Serotypes of NTS', 'Bayesian Model Results of *C. coli* and *C. jejuni'*, 'Bayesian Model Results of NTS'.

- **Are there concerns about ethical or regulatory requirements being met?
  No major concerns noted. However, the manuscript would benefit from more details about sample transport and processing conditions (e.g., average time from collection to processing), particularly to ensure confidence in microbiological integrity. Explicit mention of ethics approvals or permissions for sampling animals and accessing facilities should also be verified for completeness.**

**Answer:** We have described the details about sample transport and processing conditions.

*" All samples were transported and then processed immediately upon arrival at the laboratory of National Institute of Veterinary Research (NIVR). The time from collection to processing was within 24 hours." (lines 198-200)*

We also moved our Ethics Statement from the end of the manuscript to the method section. It is now available on lines 297-301.

*"This study was approved by the National Institute of Veterinary Research (Vietnam) (020-433/DD-YTCC) and the Royal Veterinary College (UK) Ethics and Welfare Committee (URN: 2020 1983-3). All participants informedly agreed to participate in the study. Oral consent was obtained from the farmer to sample their chickens."*

### Reviewer #2:

- **This manuscript addresses a relevant and underexplored public health issue, the distribution of foodborne pathogens, Campylobacter spp. and non-typhoidal Salmonella (NTS), along poultry production and distribution networks (PDNs) in Vietnam. The study employs a well-designed cross-sectional framework, includes key production nodes, and utilizes whole genome sequencing and Bayesian hierarchical models for prevalence estimation. These strengths position the manuscript as a valuable contribution to the field of One Health and food safety surveillance in low- and middle-income countries (LMICs). However, several methodological and analytical steps require further refinement and elaboration before publication:**

**Answer:** Thank you for your positive feedback. We responded to your queries below.

- **Sample or sample size justification is missing a rationale or calculation for the sample size (102 sites, 565 broilers) is not included. While the stratification across farms and endpoints is appreciated, it is unclear whether the study was powered to detect meaningful differences between** **site types, provinces, or broiler types. A formal justification — whether based on expected prevalence, confidence intervals, or DIC stability in Bayesian modeling — would improve methodological rigor.**

**Answer:** Thank you for highlighting this. We included a section on Power analysis in the Material and Methods (lines 387-390), in the Results (lines 431-432) and the discussion (526-529). Please refer to our detailed response to reviewer 1, point 3, above for more details.

- **Differences of sampling between pathogens: Campylobacter was detected from cecal content of birds, while NTS was identified from environmental samples. The could mislead interpretations when comparing prevalences, this limitations should be assessed explicitly.**

**Answer:** We thank the reviewer for their comment**.** NTS is usually not excreted in large amounts. They are difficult to detect without a flock-level strategy. We now state this limitation in the discussion (lines 513-516)

*"Our study has some limitations. Firstly, our model building method differed between Campylobacter spp. and NTS, as it is difficult to detect the latter at the individual level [52]. Therefore, it is not possible to assess the individual prevalence of NTS in broilers, nor to compare Campylobacter prevalence and NTS contamination probability."*

[52] Carrique-Mas JJ, Davies RH. Sampling and bacteriological detection of *Salmonella* in poultry and poultry premises: a review. Rev Sci Tech OIE. 2008;27: 665–677. doi:10.20506/rst.27.3.1829

- **It is not clear why WGS was not performed for Campylobacter: the lack of this data is an important limitation. Authors should clarify whether Campy isolates were excluded from sequencing due to logistical reasons, and justify in methods.**

**Answer:** We acknowledge the reviewer’s comment regarding the absence of whole-genome sequencing (WGS) data for *Campylobacter* isolates. This paper is an epidemiological study and not a genomic surveillance paper. We reported WGS methodology for NTS in order to identify the detected serotypes. Full WGS cross-countries analyses for *Campylobacter* and NTS isolates will be reported in two separate publications. We clarified the objectives:

"*This study aimed to estimate the prevalence of Campylobacter spp. (C. coli and C. jejuni) and NTS in broiler production and distribution networks (PDNs) using a Bayesian approach. It also investigated the NTS serotypes circulating in these PDN*" (lines 55–56)

"*NTS serotypes circulating in these PDNs were also identified*" (lines 132)

- **Limited data for farm contexts for modeling: Bayesian models incorporate site type and provincial location but omit farm-level variables that could be critical in explaining heterogeneity (density, antibiotic use, etc.). If such data were unavailable, this should be clearly stated (and addressed in limitations). If collected but not used, authors should explain the reason for exclusion in the model.**

**Answer:** Thanks for pointing this out. The objective of the study was to look at the overall prevalence across different types of sites and the variability between farms and endpoints. There was no objective to look at farm-level risk factors. Biosecurity measures and site characteristics (farm-level variables) have been collected in our study; however, these variables are of a different nature than the endpoint-level variables. Risk factor analyses of these factors would need to create separate models. We added this limitation in the discussion:

*"Additionally, other farm-level and endpoint-level risk factors were not assessed, as they differed in nature between farms and endpoints."*(lines 521-522)

## Results

### Reviewer #1:

- **Does the analysis presented match the analysis plan?
  Partially. The analyses performed are consistent with what is described in the Methods section (e.g., prevalence estimation, molecular characterization, Bayesian analysis). However, the order in which the analyses are presented in the Results does not match the order in the Methods, which affects clarity. For example, molecular analysis appears first in the Methods, but is reported after the Bayesian results in the Results section. Aligning the order between sections would improve readability**

**Answer:** Thank you for pointing this out. As mentioned above, we reorganized the flow of the Results section to improve clarity.

- **Are the results clearly and completely presented?
  Partially. While the overall findings are presented with adequate detail, some elements are unclear or not fully explained. For instance, the total number of slaughterhouses and other site types sampled is not clearly described until readers reach Table 2. This should be clarified earlier in the text or in a summary table. Additionally, the organization of some results could be improved for transparency.**

**Answer:** Thank you for pointing this out; we have now added detailed justification for site selection in the Materials and Methods, and a supplemental table (S1 Table) to include these numbers.

**Are the figures (Tables, Images) of sufficient quality for clarity?
No, revisions are needed. Several tables require reformatting for clarity:**

- **Table 3 is confusing, as the categories are not mutually exclusive and the mix of fast- and slow-growing chickens introduces an additional layer that should be subdivided for clarity. The use of asterisks is also redundant with the caption and would benefit from clearer explanation.**

**Answer:** Thank you for pointing this out. We changed Table 3.

- **Table 1 includes redundant information in the "Target" and "Product size" columns and should be simplified for better understanding.
  Overall, tables need formatting adjustments and clearer labeling to enhance their interpretability.**

**Answer:** We have changed Table 1 as suggested.

### Reviewer #2:

- **Authors present detailed descriptive and model-based findings on the prevalence of Campylobacter spp. and non-typhoidal Salmonella (NTS) across different poultry production and distribution sites in northern Vietnam. While the structure is clear and the use of Bayesian high density intervals is appropriate, there are several areas that require clarification: The manuscript frequently alternates between reporting crude (observed) prevalence and modeled posterior estimates without always making the distinction clear. This could lead to confusion (for posterior less familiar readers with Bayesian models). It would help to clearly label whether a prevalence figure is from raw data or model output in both text and tables.**

**Answer:** The reviewer made a valid point, and we concur. We have specified with observed and posterior estimate of prevalence at different places in the manuscript:

*“Table 2. The number and proportions of chicken samples testing positive by chicken types and by site types for Campylobacter spp.* *(observed data)." (lines 311–312)*

*“Table 3. The number and proportion of chicken samples testing positive by site provinces for Campylobacter spp. (observed data).” (lines 318-319)*

*“Table 4. The number and percentage of environmental samples testing positive in different sites for NTS (observed data).” (lines 333–334)*

*“Table 7. Probability of site-level and bird-level C. coli occurrence in different sites and chicken types (mode; odds ratio [OR], plus 95% High-Density Interval (HDI)* *(model-based posterior estimates).” (lines 392–393)*

*“Table 8. Probability of site-level and bird-level of C. jejuni occurrence in different sites and chicken types (mode; odds ratio [OR], plus 95% High Density Interval (HDI)) (model-based posterior estimates).” (lines 395–397)*

*“Table 10. The probability of environmental sample testing positive for Nontyphoidal Salmonella in farms and endpoint (model-based posterior estimates).” (lines 434–435)*

- **Integration of genomic data: I strongly suggest a deeper genomic analysis (only NTS serotype diversity was summarized). Authors should align with their aims why more extensive genomic analysis was not performed. This should be clarified in the discussion and considered a missed opportunity.**

**Answer:** We understand the request of the reviewer. However, as explained above, the genomic analysis is not in the scope of this paper.

- **Model interpretations are not detailed: authors state that convergence was achieved and effective sample sizes were sufficient, but diagnostic results (e.g., trace plots, Rhat, PPC summaries) are only described in supplementary materials. Including a brief narrative interpretation will help.**

**Answer:** Thank you for pointing this out; We added short descriptions to complete the diagnostic results for *Campylobacter* and NTS models.

*"The trace (n chains = 4, n iterations = 50000) of these two selected models (one for C. coli and one for C. jejuni) displayed tight, consistent horizontal bands, indicating no visual signs of non-convergence within the chains " (lines 342–344)*

*"The PPC of the selected C. jejuni model shows that posterior predictions for wholesale markets underestimated the observed proportion of C. jejuni-positive broilers (predicted: 4.1% [95% HDI 1.0–13.3%]; observed: 13.2%)" (lines 358–359)*

*“Convergence was successfully achieved for all models, with Gelman-Rubin convergency statistics below 1.01. The trace (n chains = 4, n iterations = 50000) showing consistent horizontal bands, and pairs plots for parameters of the selected NTS model are shown in S8 and S9 Figs.”(lines 412–415)*

## Conclusions

### Reviewer #1:

- **Are the conclusions supported by the data presented?
  Yes. The conclusions are generally supported by the data presented in the Results. The study finds important prevalence data on Campylobacter and non-typhoidal Salmonella in the broiler chicken production and distribution networks in Vietnam. However, some clarifications on the analysis process (particularly regarding** **site selection and sampling strategy) would further strengthen the connection between the data and the conclusions.**

**Answer:** The methods have been adapted following the reviewer and editor comments. Please refer to our previous answer in the method comments.

- **Are the limitations of the analysis clearly described?
  Partially. While the study provides a thorough analysis, the limitations are not fully addressed. The manuscript would benefit from a more explicit discussion of potential sampling biases (e.g., selection bias in site types or sample transport conditions), methodological limitations (e.g., the possibility of false negatives/positives in pathogen detection), and generalizability of the findings. A clear mention of limitations would provide a more balanced perspective on the data and its implications.**

Answer: Thanks for your suggestions. We have completed the limitation paragraph to take into account the reviewer's comment.

*"Our study has some limitations. Firstly, the modeling approaches differed between Campylobacter spp. and NTS, primarily because detecting NTS at the individual level is challenging [51]. Therefore, it is not possible to accurately assess the individual prevalence of NTS in broilers, nor to compare Campylobacter prevalence and NTS contamination probability."* (lines 513-516)

*"Additionally, other farm-level and endpoint-level risk factors were not assessed, given their heterogeneous nature across farms and endpoints. Thirdly, our site selection process may have introduced selection bias because we lacked an exhaustive list of endpoints, particularly retail markets and slaughter points. We addressed this by conducting snowball sampling interviews to identify additional eligible endpoints; however, some bias remain possible."* (lines 521-525)

*"* *Also, the observed prevalence and contamination probabilities may be underestimated. False negatives can occur due to failure in amplification during PCR or qPCR assays, even under optimal conditions. We minimized this risk by ensuring short sample transport times and validating PCR protocols rigorously."* (lines 53-537)

Reviewer #2:

**The conclusions occasionally overstate the strength of the evidence, especially where the data were limited. For example, suggesting widespread policy changes based on small sample sizes in slaughterhouses (n=6) and wholesale markets (n=10) should be more cautiously framed.**

**Answer:** We realize this limitation. This is discussed in the limitation paragraph (lines 526-529). We added a reference to this part.

- **There is no mention of limitations in the genomic analysis, particularly the lack of WGS for Campylobacter, which should temper the generalizability of genomic insights.**

**Answer:** We understand the request of the reviewer. However, as explained above, the genomic analysis is not in the scope of this paper.

- **The implications for human health are mentioned, but not linked to actual data (e.g., no integration with human case burden or clinical surveillance in Vietnam). This disconnect should be acknowledged.**

**Answer:** We respectfully disagree with the reviewer. The paper indeed focuses on the prevalence and contamination level of food-borne pathogens in the chicken food system in Vietnam. Infected chickens are not showing clinical signs in chickens. We acknowledge that we have not collected human health data, however, we strongly believe that this doesn't make this study disconnected from public health issues. We inserted the absence of human cases report in the limitation.

"*Finally, this study did not include human health surveillance data to directly link the observed pathogen contamination in chickens with human disease burden in Vietnam. However, given that contaminated chicken products are a known major source of human Campylobacter and NTS infections [2], our findings provide valuable information on the prevalence and contamination levels of foodborne pathogens in the chicken food system.” (lines 537-541)*

[2] Thomas KM, De Glanville WA, Barker GC, Benschop J, Buza JJ, Cleaveland S, et al. Prevalence of *Campylobacter* and *Salmonella* in African food animals and meat: A systematic review and meta-analysis. Int J Food Microbiol. 2020;315: 108382. doi:10.1016/j.ijfoodmicro.2019.108382

- **Conclusions are partially supported by the data: some conclusions extend beyond the strength of the evidence presented, the recommendation for broad-scale policy or surveillance interventions would be more convincing if supported by more robust genomic data (particularly for Campylobacter) or demonstrated links to human health outcomes. Generalizability to all of Vietnam should be adjusted, especially given the limited number of slaughterhouses and wholesale markets sampled.**

**Answer:** We understand the request of the reviewer. However, as explained above, the genomic analysis is not in the scope of this paper. We added the absence of human health data in the limitation (lines 537 to 541). Moreover, our conclusion generalized our results to Northern Vietnam only. We specified in the title 'Northern Vietnam".

- **Limitations of analysis are not fully described. While some are acknowledged, several important ones are underreported or omitted, including:** **asymmetric sampling approach between pathogens, absence for WGS data for Campy**

**Answer:** We have reviewed and completed the limitation paragraph to take into account both reviewers' comments on limitation (lines 512-540)

- **The manuscript acknowledges the importance of evaluating pathogen prevalence at multiple nodes in PDNs, which contributes the understanding of where contamination risks are concentrated. The authors also note the utility of molecular methods for surveillance in resource-limited settings. However, genomic component is underdeveloped, the study could have advanced understanding of Salmonella epidemiology more deeply through comparative genomics, resistance gene analysis, or clustering. For Campylobacter, the potential to advance understanding is not fully realized given the absence of WGS or deeper typing beyond species identification.**

**Answer:** Please refer to our answer to the absence of WGS questions above.

- **The manuscript appropriately frames Campylobacter and NTS as foodborne pathogens of global health concern, and highlights the potential for transmission along poultry value chains. The conclusion references the need for improved hygiene and regulation at slaughter and retail points.**

**Answer:** Thank you.

## Summary and General Comments

### Reviewer #1:

- **The manuscript titled "Prevalence of Campylobacter and non-typhoidal Salmonella along broiler chicken production and distribution networks, Vietnam" presents a well-executed and scientifically relevant study that contributes valuable insights to the global understanding of foodborne pathogens in poultry production chains. The findings are important for both the scientific community and public health authorities, particularly in the context of food safety in low- and middle-income countries. The methodology is generally sound, and the data are robust. However, several minor issues should be addressed to improve the clarity and transparency of the manuscript.**
- **Minor Comments for Revision.**
- **Objectives in the Introduction. The stated objectives at the end of the Introduction appear incomplete. While the study includes molecular characterization through sequencing, this important component is not mentioned in the stated aims. It is recommended that the authors revise the objectives to fully reflect all key aspects of the study.**

**Answer:** We modified the objectives in the abstract (lines 55–56) and in the introduction (lines 132). Sequencing was not part of the objectives.

- **Site Selection Description. The subsection "Site selection" in the Materials and Methods is somewhat confusing and lacks alignment with the first section of the Results. For instance, lines 149–150 mention: "All wholesale markets (N = 10) and slaughterhouses (N = 6) that processed slow-growing broiler chickens in the four study provinces were recruited," yet the total number of slaughterhouses sampled remains unclear until Table 2, where 18 positive samples are reported. A clearer explanation of how many slaughterhouses and other site types were evaluated should be included upfront. Consider providing a descriptive table in the Methods section to summarize the distribution and number of sites per type, and clarify whether any stratification approach was applied in the site selection process.**

**Answer:** We modified the methods in lines 162 to 176 to align with the comment. As we already have 8 tables in the paper, we placed a descriptive table in the appendices (S1 Table).

- **Method-Results Alignment. The order of methodological descriptions does not match the order of presentation in the Results section. For example, molecular analyses are presented before Bayesian analysis in the Methods, but this order is reversed in the Results. It is recommended to align the structure of the Methods with the flow of the Results to improve readability.**

**Answer:** The Material and Methods structure has been reorganized to align with the Results structure.

- **Table 3 Clarity and Formatting: Table 3 is difficult to interpret. The categories presented are not mutually exclusive, and the combination of fast- and slow-growing chicken types introduces an additional layer of categorization that could benefit from clearer subdivision. It appears that the table format may not fully conform to journal standards, and a reorganization could greatly improve comprehension. The asterisk notation used in the table is redundant with the caption and would be more useful if better integrated into a clearer explanation within the caption.**

**Answer:** Table 3 was modified according to the previous comment.

- **. Table 1 Simplification Table 1 also requires formatting improvements. The columns “Target” and “Product size” contain redundant information that could be simplified for clarity.**

**Answer:** Table 1 was adapted accordingly.

- **Sample Processing Time (Line 177). The manuscript states that "Cecal content samples were processed upon arrival at the laboratory of the National Institute of Veterinary Research." It is important to clarify the average time between sample collection and processing, and whether there was significant variability among samples, as this can influence the reliability of microbial detection.**

**Answer:** The average time is now specified in lines 198 to 200.

*" All samples were transported and then processed immediately upon arrival at the laboratory of National Institute of Veterinary Research (NIVR). The time from collection to processing is within 24 hours. “*

### Reviewer #2:

- **This manuscript presents a cross-sectional, multi-site study investigating the prevalence of Campylobacter spp. and non-typhoidal Salmonella (NTS) along poultry production and distribution networks (PDNs) in Vietnam.
  However, there are several important limitations that reduce the overall impact and scientific value of the work in its current form.**
- **Limited genomic analysis: WGS was performed on Salmonella isolates but only used for serotyping . There is no analysis of resistance genes, virulence genes, or genomic clustering/phylogeny. These elements are widely expected in genomic surveillance studies and would enhance the public health relevance of the findings. Moreover, Campylobacter isolates were not sequenced, and this gap is not adequately addressed in the text.**

**Answer:** These comments are addressed above.

- **No explicit hypotheses tested and limited statistical interpretation: the study is descriptive, and although Bayesian modeling is correctly implemented, the lack of posterior probability estimates of differences between categories weakens interpretability. For example, the manuscript does not quantify how likely it is that prevalence differs between site types or between species (Campy coli vs C. jejuni), despite having the framework to do so. Authors should report posterior probabilities of group differences where relevant, clarifying differences supported by data.**

**Answer:**

We appreciate the reviewer's suggestion to leverage the full potential of our Bayesian framework. However, Bayesian analysis provides a framework for quantifying uncertainty about parameter values, rather than simply making binary decisions about rejecting or failing to reject a hypothesis [1]. Although Bayes factors can be used for comparing hypotheses, the core of Bayesian analysis lies in updating beliefs about parameters rather than accepting or rejecting a single null hypothesis [1,2]. The differences between site types or other factors can be seen with the HDI.

[1] Shikano S. Hypothesis testing in the Bayesian framework. Swiss Polit Sci Rev. 2019;25: 288–299. doi:10.1111/spsr.12375

[2] Fornacon-Wood I, Mistry H, Johnson-Hart C, Faivre-Finn C, O’Connor JPB, Price GJ. Understanding the Differences Between Bayesian and Frequentist Statistics. Int J Radiat Oncol Biol Phys. 2022;112: 1076–1082. doi:10.1016/j.ijrobp.2021.12.011

**Some critical limitations are only partially acknowledged. For example, the asymmetric sampling strategy (Campy from cecal samples; Salmonella from environmental swabs) is not discussed, and the limited genomic scope for Campylobacter is omitted entirely. Conclusions imply policy recommendations that are not fully supported by the dataset (e.g., generalizable surveillance implications from very few slaughterhouses sampled).**

**Answer:** The limitation paragraph was completed to address these comments. Regarding absence for WGS data for Campy. As explained above, the genomic analysis is not in the scope of this paper. We clarified this in our objective.
